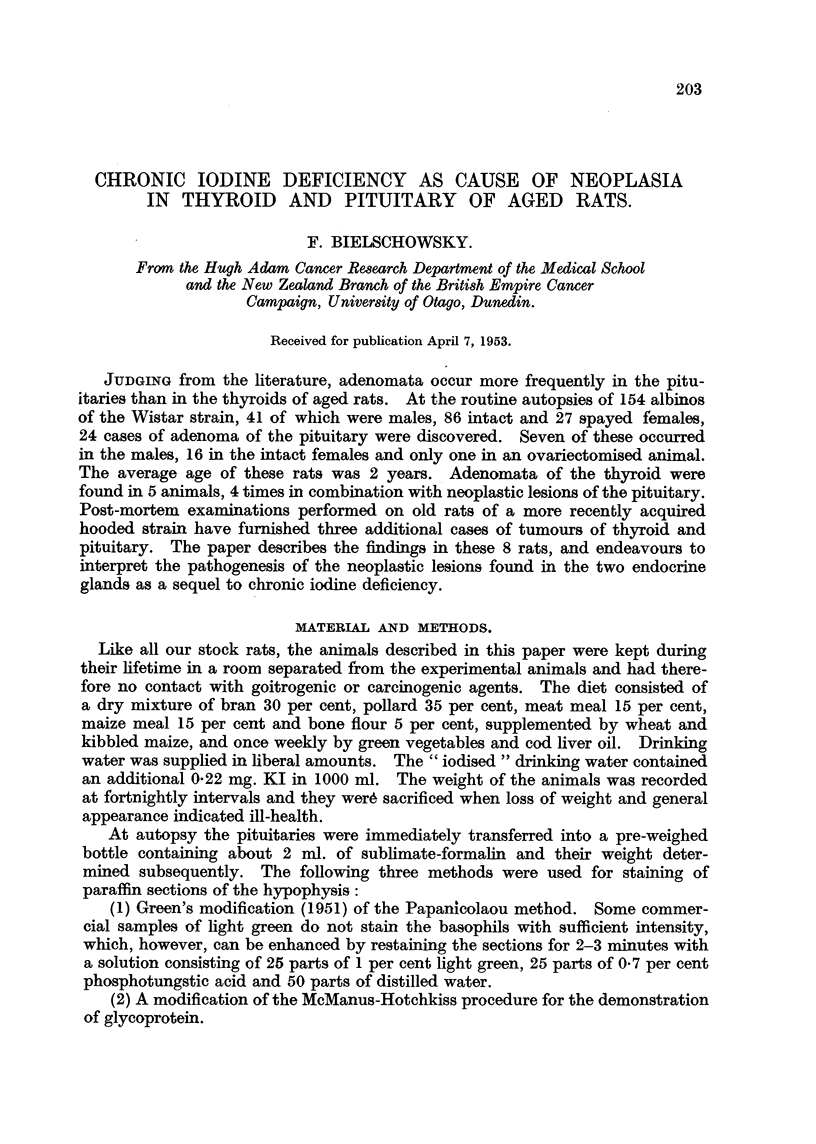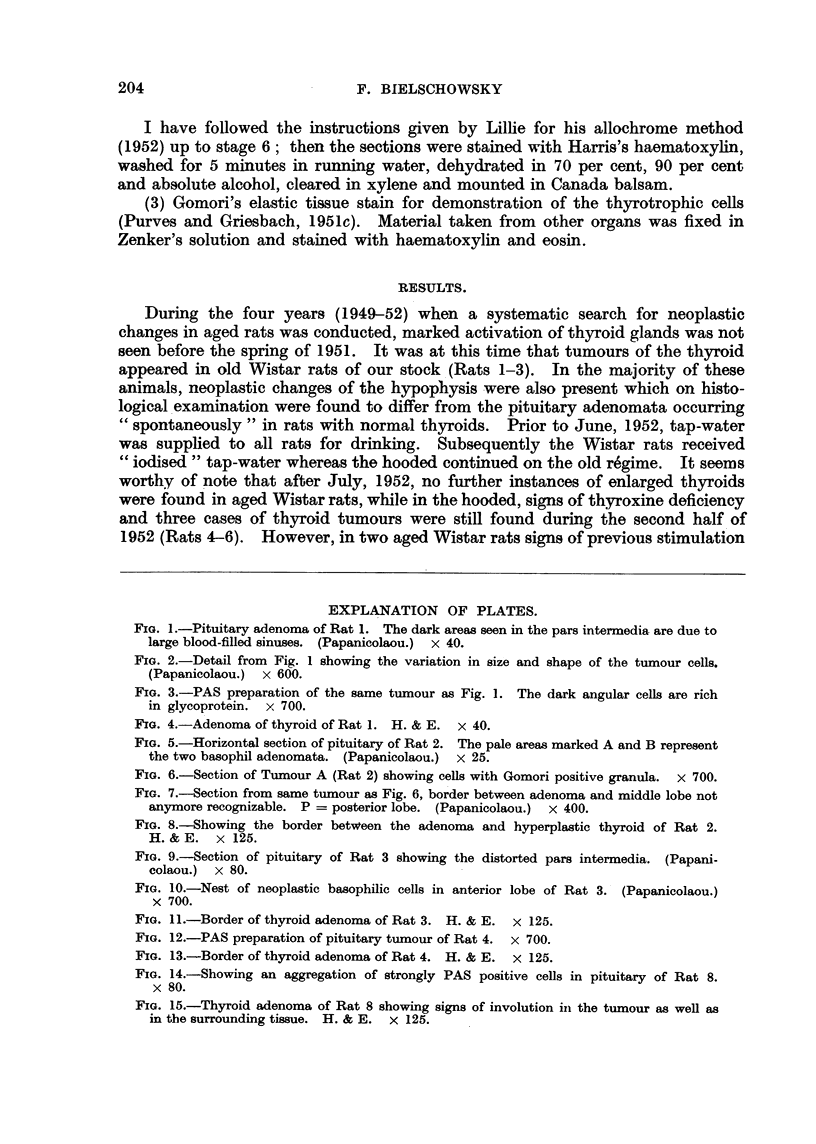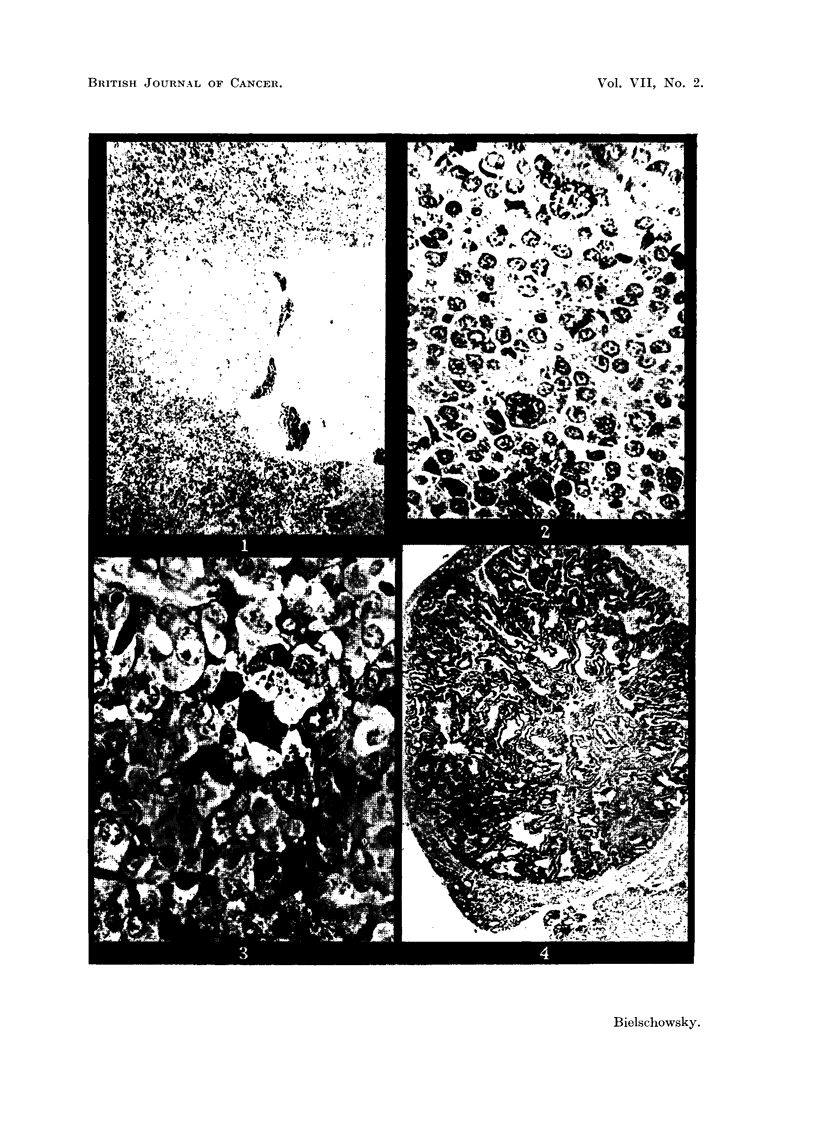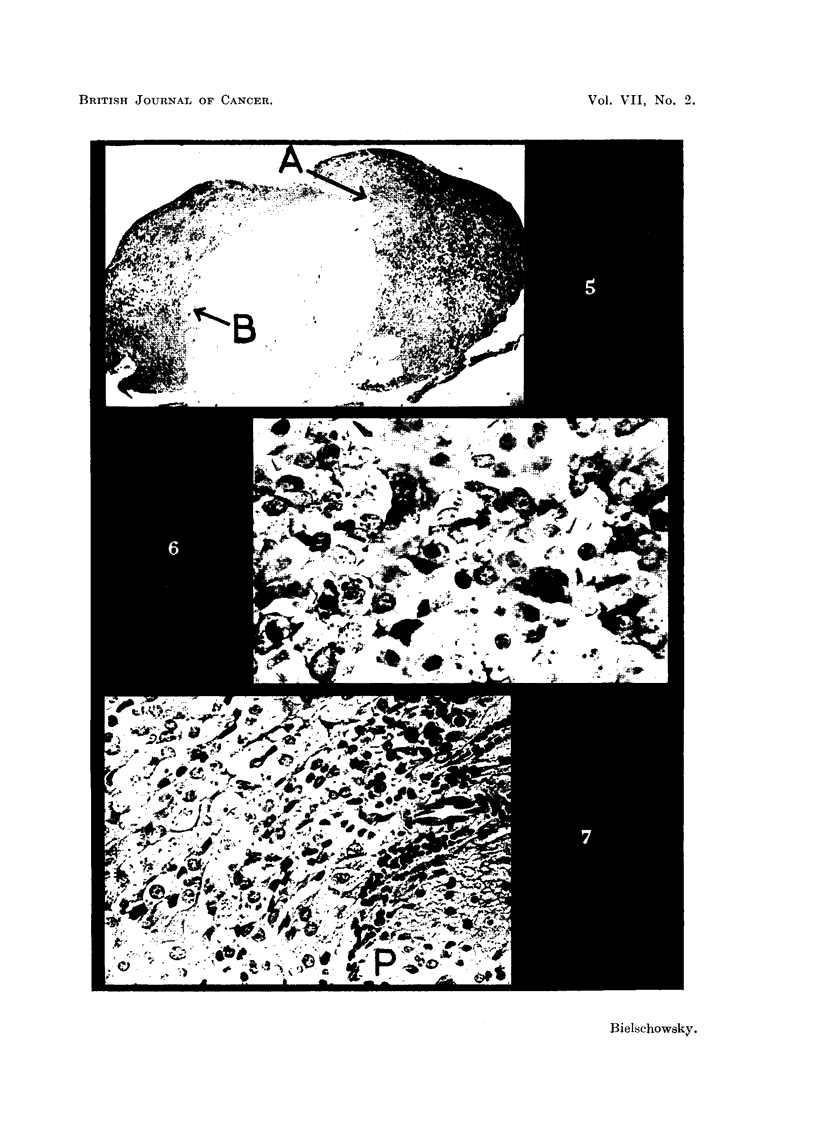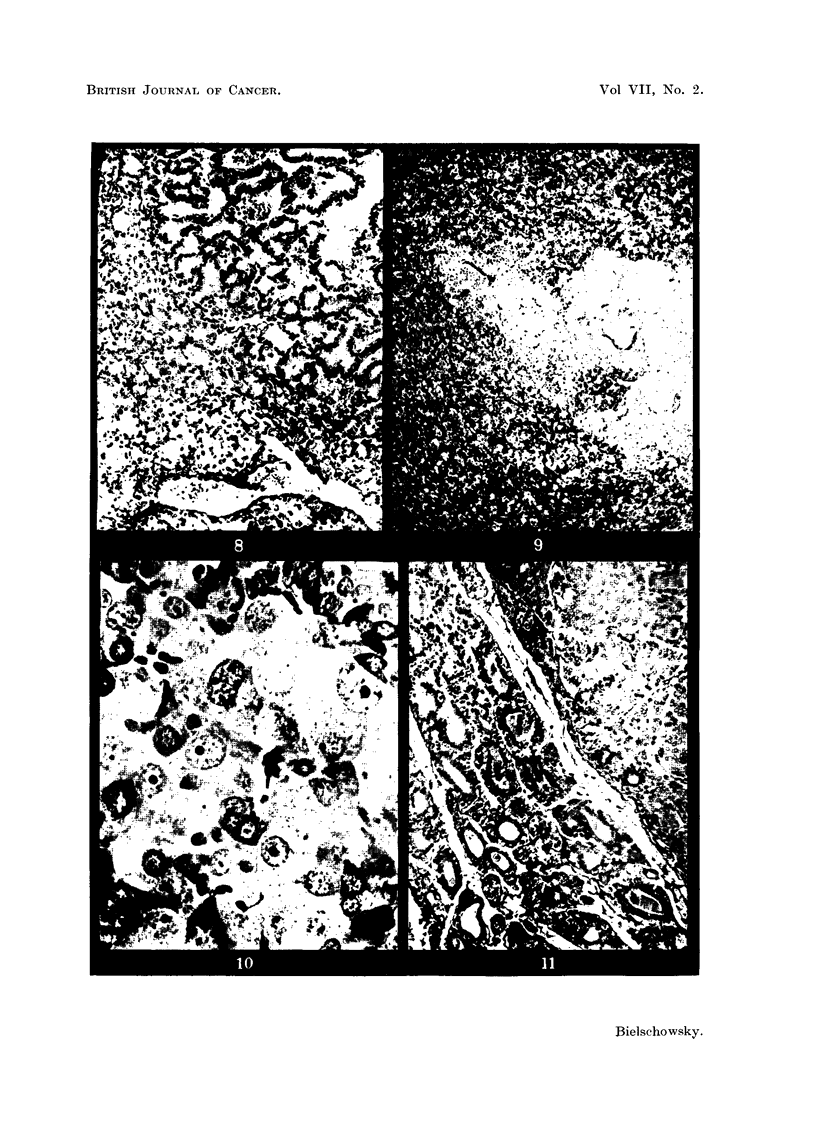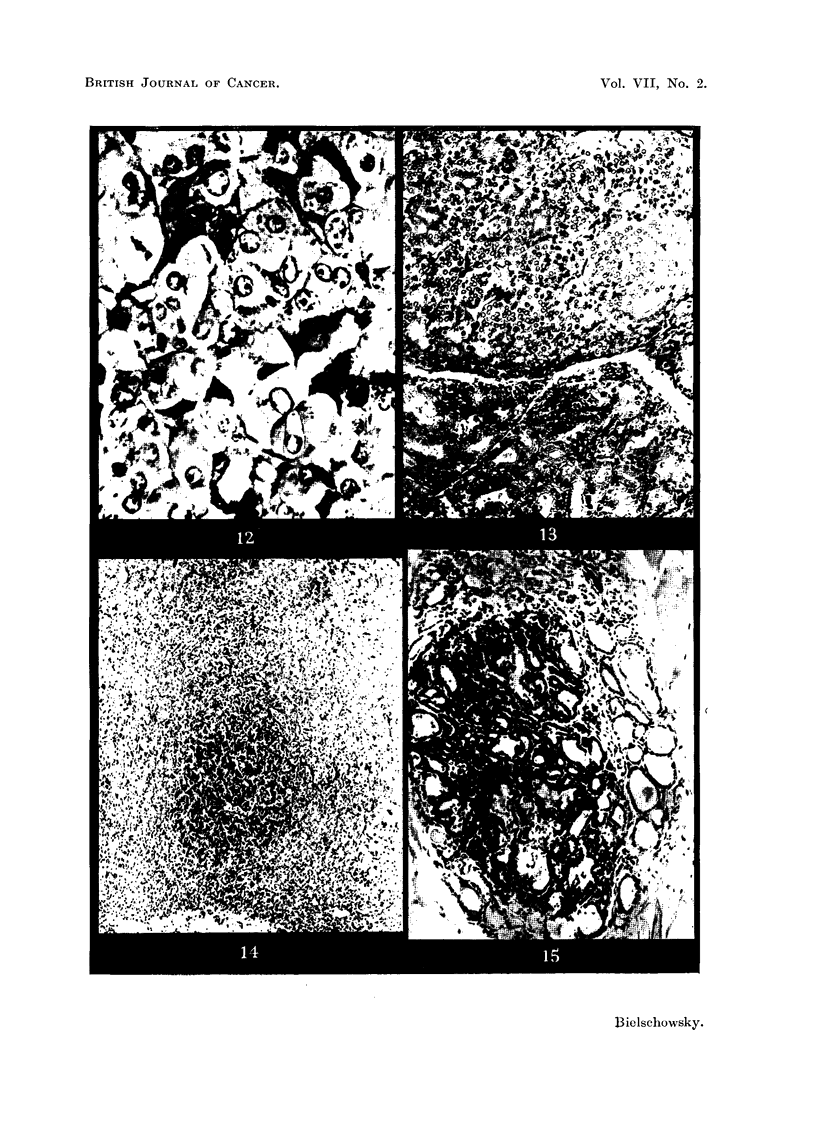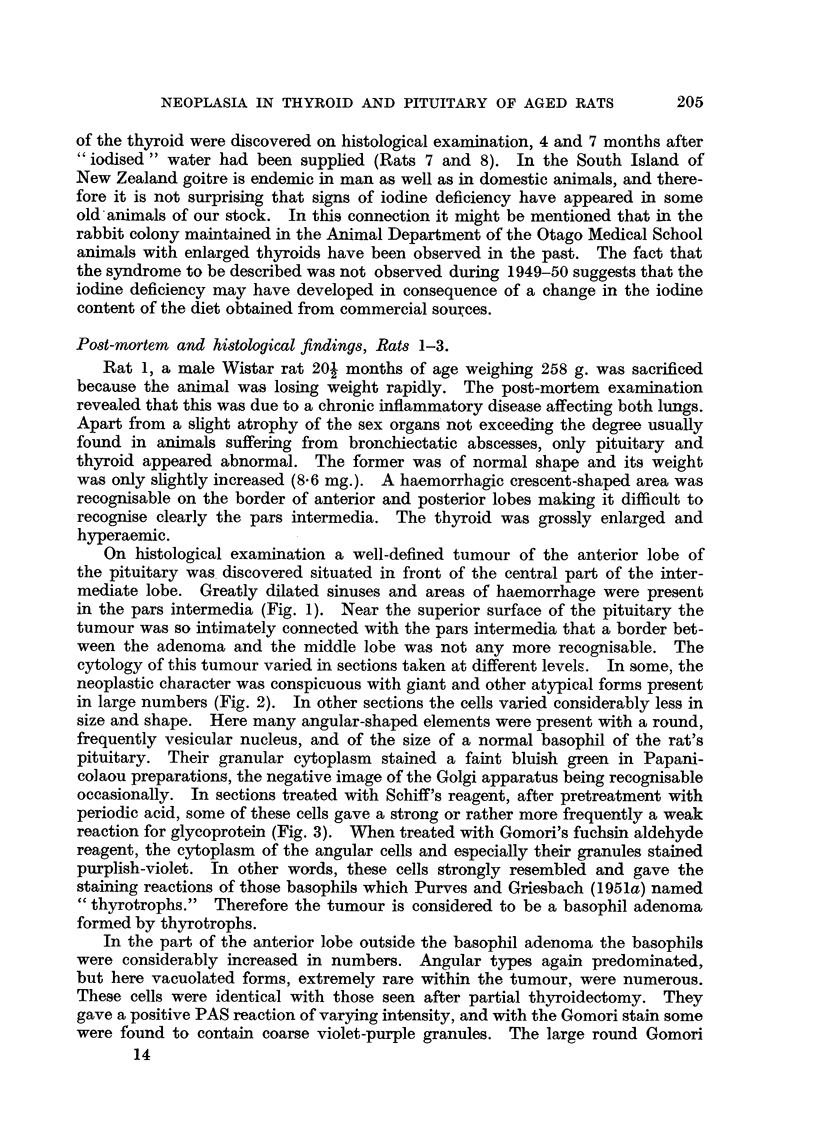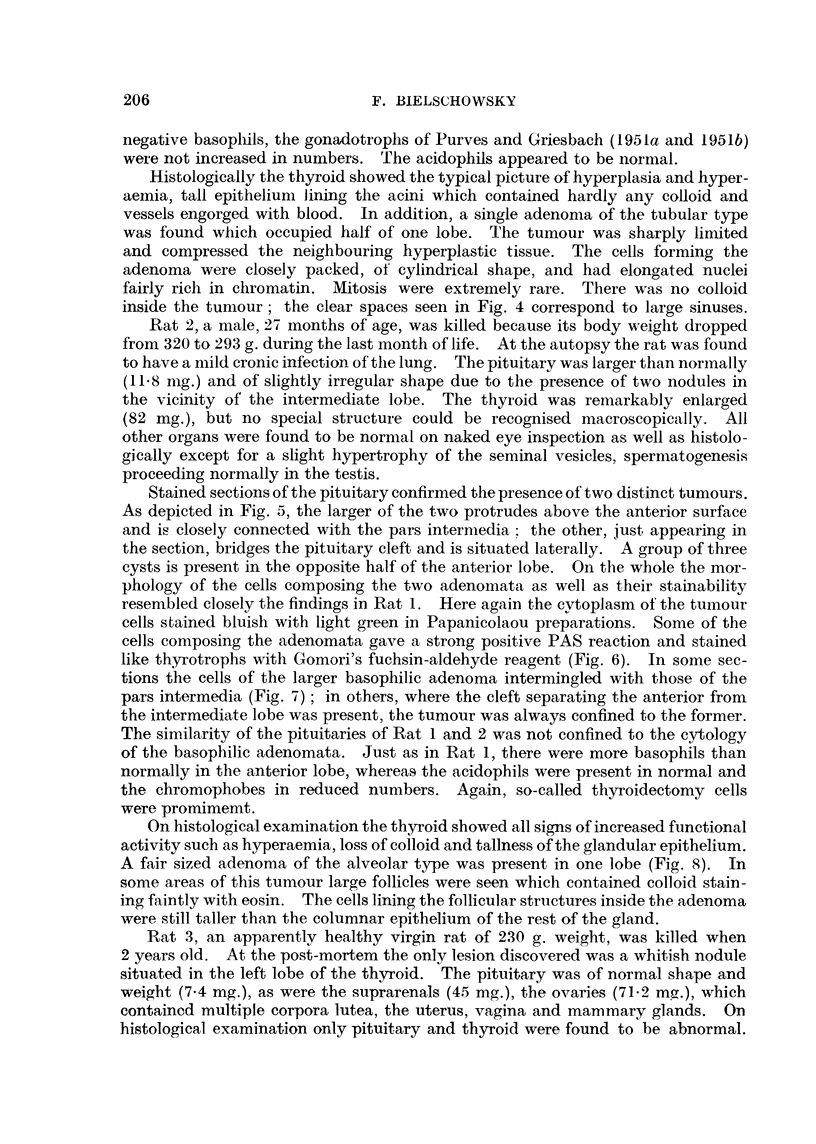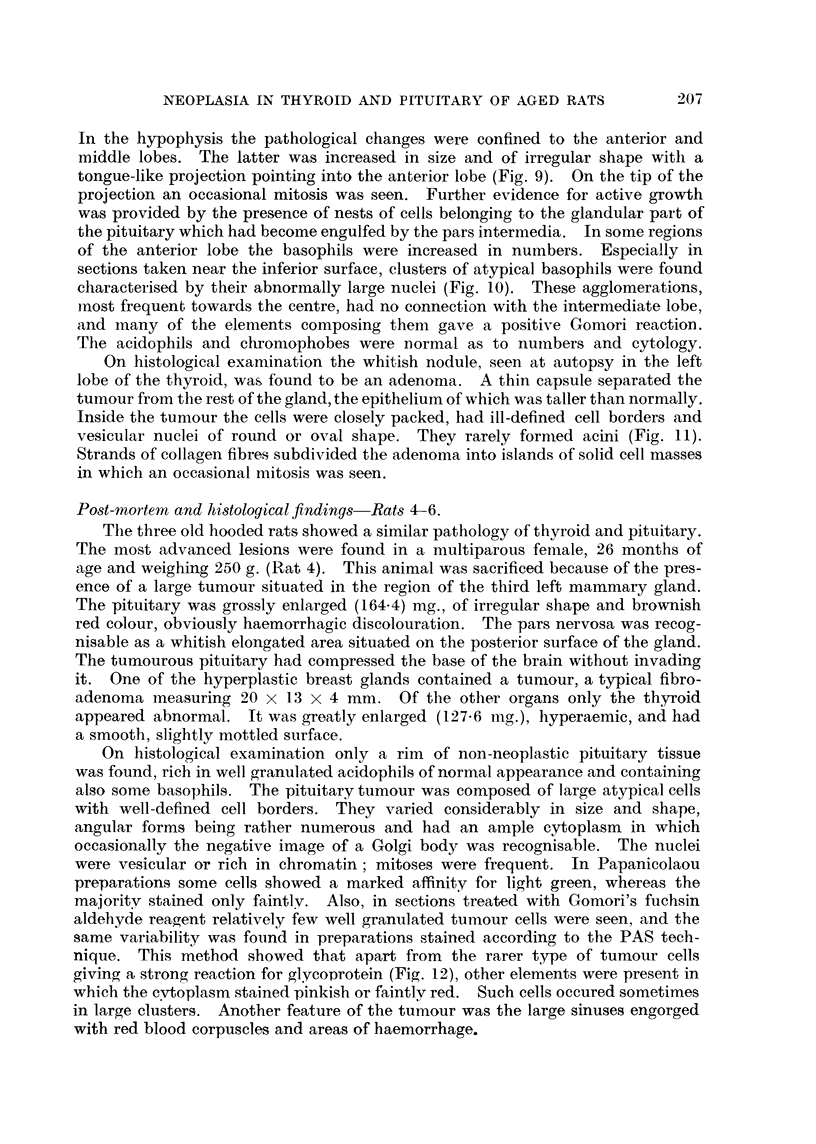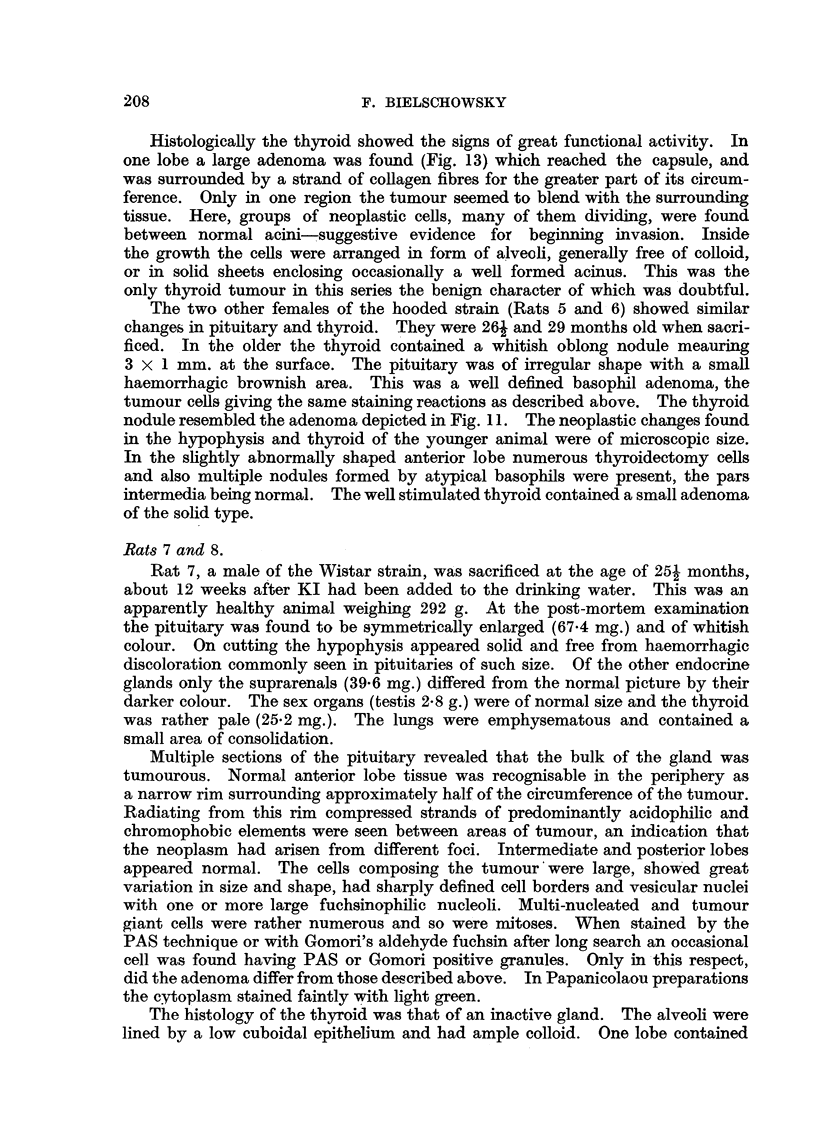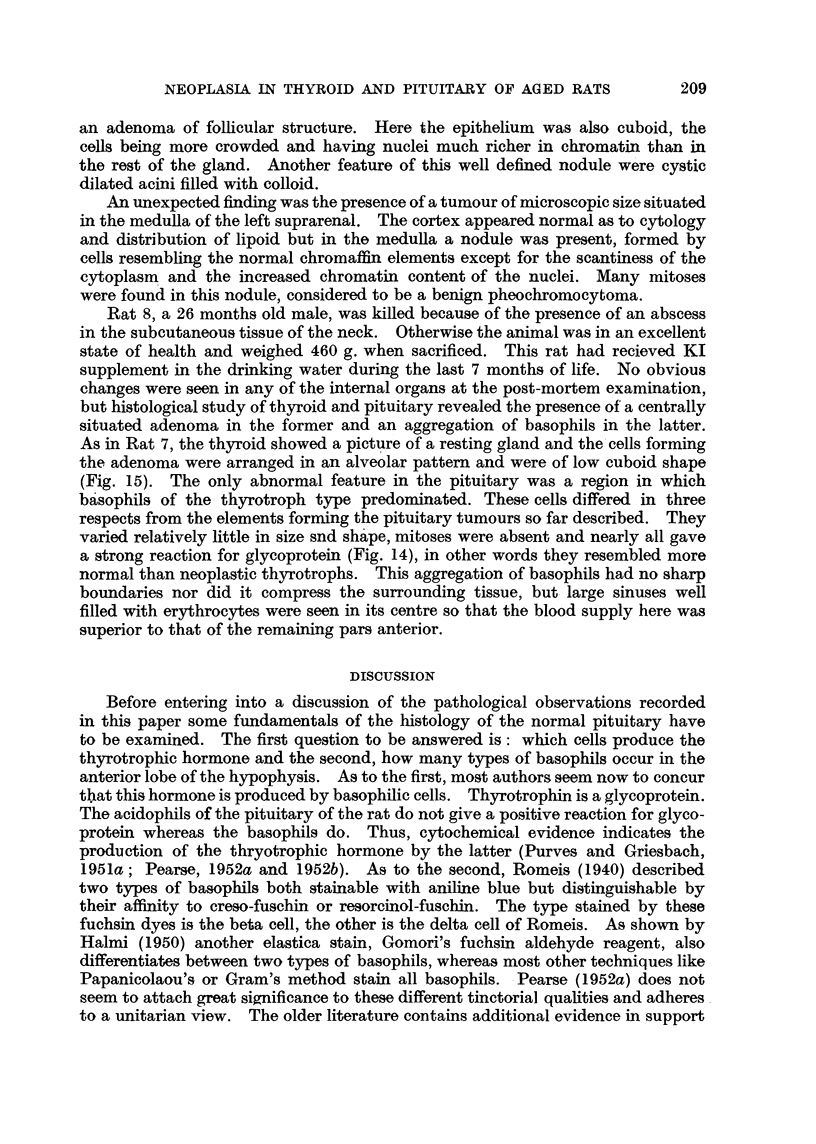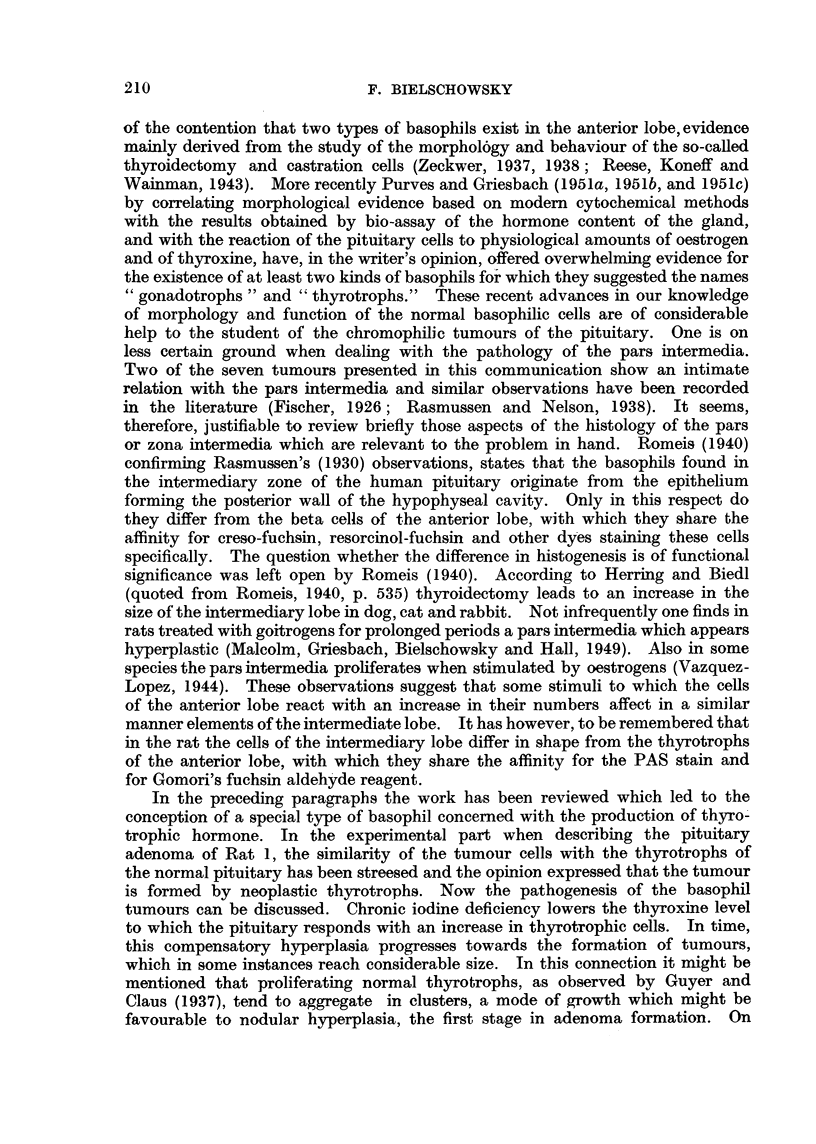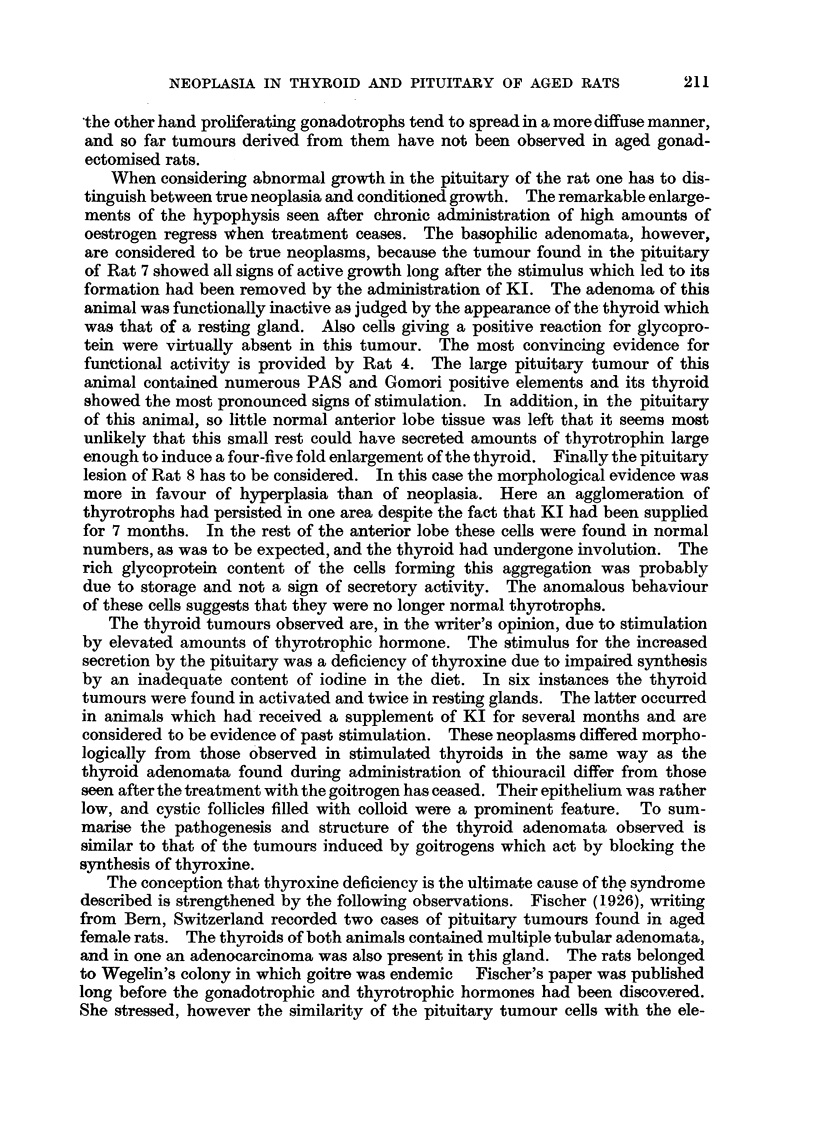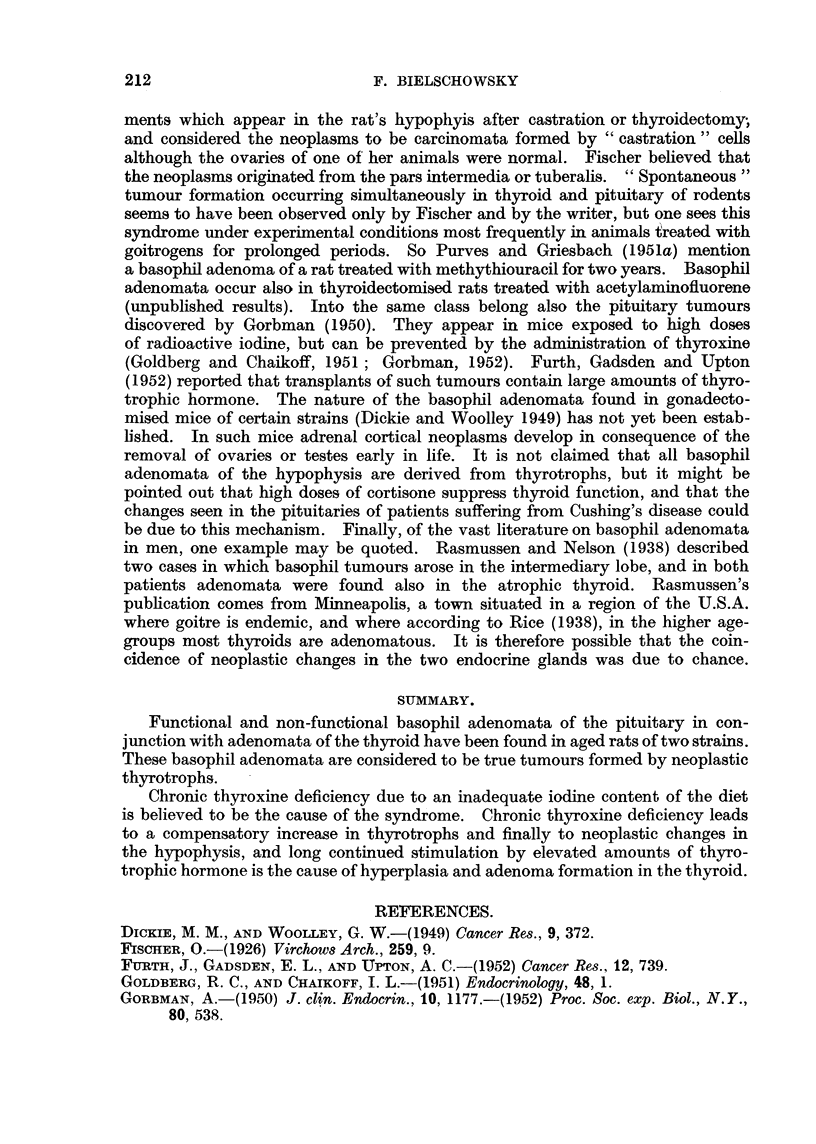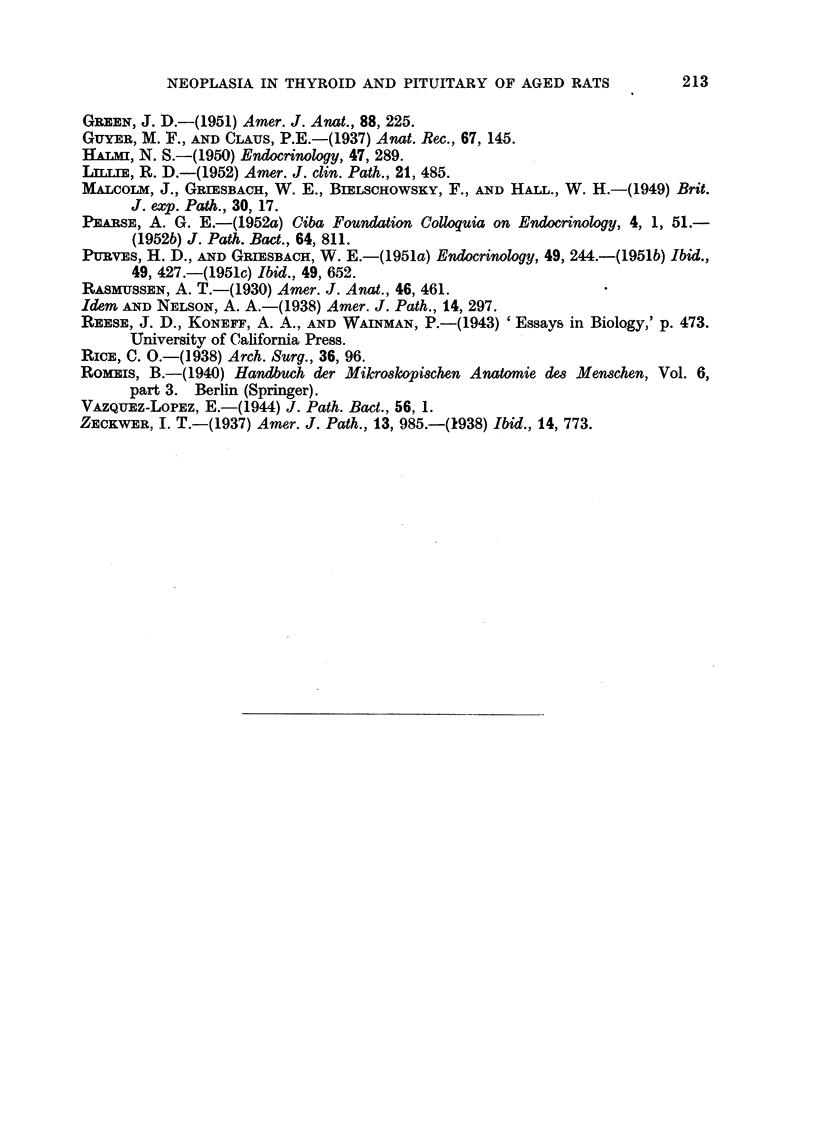# Chronic Iodine Deficiency as Cause of Neoplasia in Thyroid and Pituitary of Aged Rats

**DOI:** 10.1038/bjc.1953.18

**Published:** 1953-06

**Authors:** F. Bielschowsky

## Abstract

**Images:**


### CHRONIC IODINE DEFICIENCY AS CAUSE OF NEOPLASIA IN THYROID AND PITUITARY OF AGED RATS.

F. BIELSCHOWSKY.

*From the Hugh Adam Cancer Research Department of the Medical School and the New Zealand Branch of the British Empire Cancer Campaign, University of Otago, Dunedin.*

Received for publication April 7, 1953.

JUDGING from the literature, adenomata occur more frequently in the pituitaries than in the thyroids of aged rats. At the routine autopsies of 154 albinos of the Wistar strain, 41 of which were males, 86 intact and 27 spayed females, 24 cases of adenoma of the pituitary were discovered. Seven of these occurred in the males, 16 in the intact females and only one in an ovariectomised animal. The average age of these rats was 2 years. Adenomata of the thyroid were found in 5 animals, 4 times in combination with neoplastic lesions of the pituitary. Post-mortem examinations performed on old rats of a more recently acquired hooded strain have furnished three additional cases of tumours of thyroid and pituitary. The paper describes the findings in these 8 rats, and endeavours to interpret the pathogenesis of the neoplastic lesions found in the two endocrine glands as a sequel to chronic iodine deficiency.

### MATERIAL AND METHODS.

Like all our stock rats, the animals described in this paper were kept during their lifetime in a room separated from the experimental animals and had therefore no contact with goitrogenic or carcinogenic agents. The diet consisted of a dry mixture of bran 30 per cent, pollard 35 per cent, meat meal 15 per cent, maize meal 15 per cent and bone flour 5 per cent, supplemented by wheat and kibbled maize, and once weekly by green vegetables and cod liver oil. Drinking water was supplied in liberal amounts. The " iodised " drinking water contained an additional 0·22 mg. KI in 1000 ml. The weight of the animals was recorded at fortnightly intervals and they were sacrificed when loss of weight and general appearance indicated ill-health.

At autopsy the pituitaries were immediately transferred into a pre-weighed bottle containing about 2 ml. of sublimate-formalin and their weight determined subsequently. The following three methods were used for staining of paraffin sections of the hypophysis :

(1) Green's modification (1951) of the Papanicolaou method. Some commercial samples of light green do not stain the basophils with sufficient intensity, which, however, can be enhanced by restaining the sections for 2–3 minutes with a solution consisting of 25 parts of 1 per cent light green, 25 parts of 0·7 per cent phosphotungstic acid and 50 parts of distilled water.

(2) A modification of the McManus-Hotchkiss procedure for the demonstration of glycoprotein.

I have followed the instructions given by Lillie for his allochrome method (1952) up to stage 6 ; then the sections were stained with Harris's haematoxylin, washed for 5 minutes in running water, dehydrated in 70 per cent, 90 per cent and absolute alcohol, cleared in xylene and mounted in Canada balsam.

(3) Gomori's elastic tissue stain for demonstration of the thyrotrophic cells (Purves and Griesbach, 1951*c*). Material taken from other organs was fixed in Zenker's solution and stained with haematoxylin and eosin.

### RESULTS.

During the four years (1949–52) when a systematic search for neoplastic changes in aged rats was conducted, marked activation of thyroid glands was not seen before the spring of 1951. It was at this time that tumours of the thyroid appeared in old Wistar rats of our stock (Rats 1–3). In the majority of these animals, neoplastic changes of the hypophysis were also present which on histological examination were found to differ from the pituitary adenomata occurring "spontaneously" in rats with normal thyroids. Prior to June, 1952, tap-water was supplied to all rats for drinking. Subsequently the Wistar rats received "iodised" tap-water whereas the hooded continued on the old régime. It seems worthy of note that after July, 1952, no further instances of enlarged thyroids were found in aged Wistar rats, while in the hooded, signs of thyroxine deficiency and three cases of thyroid tumours were still found during the second half of 1952 (Rats 4–6). However, in two aged Wistar rats signs of previous stimulation

---

### EXPLANATION OF PLATES.

FIG. 1.—Pituitary adenoma of Rat 1. The dark areas seen in the pars intermedia are due to large blood-filled sinuses. (Papanicolaou.) × 40.

FIG. 2.—Detail from Fig. 1 showing the variation in size and shape of the tumour cells. (Papanicolaou.) × 600.

FIG. 3.—PAS preparation of the same tumour as Fig. 1. The dark angular cells are rich in glycoprotein. × 700.

FIG. 4.—Adenoma of thyroid of Rat 1. H. & E. × 40.

FIG. 5.—Horizontal section of pituitary of Rat 2. The pale areas marked A and B represent the two basophil adenomata. (Papanicolaou.) × 25.

FIG. 6.—Section of Tumour A (Rat 2) showing cells with Gomori positive granula. × 700.

FIG. 7.—Section from same tumour as Fig. 6, border between adenoma and middle lobe not anymore recognizable. P = posterior lobe. (Papanicolaou.) × 400.

FIG. 8.—Showing the border between the adenoma and hyperplastic thyroid of Rat 2. H. & E. × 125.

FIG. 9.—Section of pituitary of Rat 3 showing the distorted pars intermedia. (Papanicolaou.) × 80.

FIG. 10.—Nest of neoplastic basophilic cells in anterior lobe of Rat 3. (Papanicolaou.) × 700.

FIG. 11.—Border of thyroid adenoma of Rat 3. H. & E. × 125.

FIG. 12.—PAS preparation of pituitary tumour of Rat 4. × 700.

FIG. 13.—Border of thyroid adenoma of Rat 4. H. & E. × 125.

FIG. 14.—Showing an aggregation of strongly PAS positive cells in pituitary of Rat 8. × 80.

FIG. 15.—Thyroid adenoma of Rat 8 showing signs of involution in the tumour as well as in the surrounding tissue. H. & E. × 125.

Bielschowsky.

5

6

7

Bielschowsky.

8

9

10

11

Bielschowsky.

12

13

14

15

Bielschowsky.

of the thyroid were discovered on histological examination, 4 and 7 months after "iodised" water had been supplied (Rats 7 and 8). In the South Island of New Zealand goitre is endemic in man as well as in domestic animals, and therefore it is not surprising that signs of iodine deficiency have appeared in some old animals of our stock. In this connection it might be mentioned that in the rabbit colony maintained in the Animal Department of the Otago Medical School animals with enlarged thyroids have been observed in the past. The fact that the syndrome to be described was not observed during 1949–50 suggests that the iodine deficiency may have developed in consequence of a change in the iodine content of the diet obtained from commercial sources.

*Post-mortem and histological findings, Rats* 1–3.

Rat 1, a male Wistar rat 20½ months of age weighing 258 g. was sacrificed because the animal was losing weight rapidly. The post-mortem examination revealed that this was due to a chronic inflammatory disease affecting both lungs. Apart from a slight atrophy of the sex organs not exceeding the degree usually found in animals suffering from bronchiectatic abscesses, only pituitary and thyroid appeared abnormal. The former was of normal shape and its weight was only slightly increased (8·6 mg.). A haemorrhagic crescent-shaped area was recognisable on the border of anterior and posterior lobes making it difficult to recognise clearly the pars intermedia. The thyroid was grossly enlarged and hyperaemic.

On histological examination a well-defined tumour of the anterior lobe of the pituitary was discovered situated in front of the central part of the intermediate lobe. Greatly dilated sinuses and areas of haemorrhage were present in the pars intermedia (Fig. 1). Near the superior surface of the pituitary the tumour was so intimately connected with the pars intermedia that a border between the adenoma and the middle lobe was not any more recognisable. The cytology of this tumour varied in sections taken at different levels. In some, the neoplastic character was conspicuous with giant and other atypical forms present in large numbers (Fig. 2). In other sections the cells varied considerably less in size and shape. Here many angular-shaped elements were present with a round, frequently vesicular nucleus, and of the size of a normal basophil of the rat's pituitary. Their granular cytoplasm stained a faint bluish green in Papanicolaou preparations, the negative image of the Golgi apparatus being recognisable occasionally. In sections treated with Schiff's reagent, after pretreatment with periodic acid, some of these cells gave a strong or rather more frequently a weak reaction for glycoprotein (Fig. 3). When treated with Gomori's fuchsin aldehyde reagent, the cytoplasm of the angular cells and especially their granules stained purplish-violet. In other words, these cells strongly resembled and gave the staining reactions of those basophils which Purves and Griesbach (1951*a*) named "thyrotrophs." Therefore the tumour is considered to be a basophil adenoma formed by thyrotrophs.

In the part of the anterior lobe outside the basophil adenoma the basophils were considerably increased in numbers. Angular types again predominated, but here vacuolated forms, extremely rare within the tumour, were numerous. These cells were identical with those seen after partial thyroidectomy. They gave a positive PAS reaction of varying intensity, and with the Gomori stain some were found to contain coarse violet-purple granules. The large round Gomori negative basophils, the gonadotrophs of Purves and Griesbach (1951*a* and 1951*b*) were not increased in numbers. The acidophils appeared to be normal.

Histologically the thyroid showed the typical picture of hyperplasia and hyperaemia, tall epithelium lining the acini which contained hardly any colloid and vessels engorged with blood. In addition, a single adenoma of the tubular type was found which occupied half of one lobe. The tumour was sharply limited and compressed the neighbouring hyperplastic tissue. The cells forming the adenoma were closely packed, of cylindrical shape, and had elongated nuclei fairly rich in chromatin. Mitosis were extremely rare. There was no colloid inside the tumour ; the clear spaces seen in Fig. 4 correspond to large sinuses.

Rat 2, a male, 27 months of age, was killed because its body weight dropped from 320 to 293 g. during the last month of life. At the autopsy the rat was found to have a mild cronic infection of the lung. The pituitary was larger than normally (11·8 mg.) and of slightly irregular shape due to the presence of two nodules in the vicinity of the intermediate lobe. The thyroid was remarkably enlarged (82 mg.), but no special structure could be recognised macroscopically. All other organs were found to be normal on naked eye inspection as well as histologically except for a slight hypertrophy of the seminal vesicles, spermatogenesis proceeding normally in the testis.

Stained sections of the pituitary confirmed the presence of two distinct tumours. As depicted in Fig. 5, the larger of the two protrudes above the anterior surface and is closely connected with the pars intermedia ; the other, just appearing in the section, bridges the pituitary cleft and is situated laterally. A group of three cysts is present in the opposite half of the anterior lobe. On the whole the morphology of the cells composing the two adenomata as well as their stainability resembled closely the findings in Rat 1. Here again the cytoplasm of the tumour cells stained bluish with light green in Papanicolaou preparations. Some of the cells composing the adenomata gave a strong positive PAS reaction and stained like thyrotrophs with Gomori's fuchsin-aldehyde reagent (Fig. 6). In some sections the cells of the larger basophilic adenoma intermingled with those of the pars intermedia (Fig. 7) ; in others, where the cleft separating the anterior from the intermediate lobe was present, the tumour was always confined to the former. The similarity of the pituitaries of Rat 1 and 2 was not confined to the cytology of the basophilic adenomata. Just as in Rat 1, there were more basophils than normally in the anterior lobe, whereas the acidophils were present in normal and the chromophobes in reduced numbers. Again, so-called thyroidectomy cells were promimemt.

On histological examination the thyroid showed all signs of increased functional activity such as hyperaemia, loss of colloid and tallness of the glandular epithelium. A fair sized adenoma of the alveolar type was present in one lobe (Fig. 8). In some areas of this tumour large follicles were seen which contained colloid staining faintly with eosin. The cells lining the follicular structures inside the adenoma were still taller than the columnar epithelium of the rest of the gland.

Rat 3, an apparently healthy virgin rat of 230 g. weight, was killed when 2 years old. At the post-mortem the only lesion discovered was a whitish nodule situated in the left lobe of the thyroid. The pituitary was of normal shape and weight (7·4 mg.), as were the suprarenals (45 mg.), the ovaries (71·2 mg.), which contained multiple corpora lutea, the uterus, vagina and mammary glands. On histological examination only pituitary and thyroid were found to be abnormal.

In the hypophysis the pathological changes were confined to the anterior and middle lobes. The latter was increased in size and of irregular shape with a tongue-like projection pointing into the anterior lobe (Fig. 9). On the tip of the projection an occasional mitosis was seen. Further evidence for active growth was provided by the presence of nests of cells belonging to the glandular part of the pituitary which had become engulfed by the pars intermedia. In some regions of the anterior lobe the basophils were increased in numbers. Especially in sections taken near the inferior surface, clusters of atypical basophils were found characterised by their abnormally large nuclei (Fig. 10). These agglomerations, most frequent towards the centre, had no connection with the intermediate lobe, and many of the elements composing them gave a positive Gomori reaction. The acidophils and chromophobes were normal as to numbers and cytology.

On histological examination the whitish nodule, seen at autopsy in the left lobe of the thyroid, was found to be an adenoma. A thin capsule separated the tumour from the rest of the gland, the epithelium of which was taller than normally. Inside the tumour the cells were closely packed, had ill-defined cell borders and vesicular nuclei of round or oval shape. They rarely formed acini (Fig. 11). Strands of collagen fibres subdivided the adenoma into islands of solid cell masses in which an occasional mitosis was seen.

*Post-mortem and histological findings—Rats* 4–6.

The three old hooded rats showed a similar pathology of thyroid and pituitary. The most advanced lesions were found in a multiparous female, 26 months of age and weighing 250 g. (Rat 4). This animal was sacrificed because of the presence of a large tumour situated in the region of the third left mammary gland. The pituitary was grossly enlarged (164·4) mg., of irregular shape and brownish red colour, obviously haemorrhagic discolouration. The pars nervosa was recognisable as a whitish elongated area situated on the posterior surface of the gland. The tumourous pituitary had compressed the base of the brain without invading it. One of the hyperplastic breast glands contained a tumour, a typical fibroadenoma measuring 20 $\times$ 13 $\times$ 4 mm. Of the other organs only the thyroid appeared abnormal. It was greatly enlarged (127·6 mg.), hyperaemic, and had a smooth, slightly mottled surface.

On histological examination only a rim of non-neoplastic pituitary tissue was found, rich in well granulated acidophils of normal appearance and containing also some basophils. The pituitary tumour was composed of large atypical cells with well-defined cell borders. They varied considerably in size and shape, angular forms being rather numerous and had an ample cytoplasm in which occasionally the negative image of a Golgi body was recognisable. The nuclei were vesicular or rich in chromatin ; mitoses were frequent. In Papanicolaou preparations some cells showed a marked affinity for light green, whereas the majority stained only faintly. Also, in sections treated with Gomori's fuchsin aldehyde reagent relatively few well granulated tumour cells were seen, and the same variability was found in preparations stained according to the PAS technique. This method showed that apart from the rarer type of tumour cells giving a strong reaction for glycoprotein (Fig. 12), other elements were present in which the cytoplasm stained pinkish or faintly red. Such cells occured sometimes in large clusters. Another feature of the tumour was the large sinuses engorged with red blood corpuscles and areas of haemorrhage.

Histologically the thyroid showed the signs of great functional activity. In one lobe a large adenoma was found (Fig. 13) which reached the capsule, and was surrounded by a strand of collagen fibres for the greater part of its circumference. Only in one region the tumour seemed to blend with the surrounding tissue. Here, groups of neoplastic cells, many of them dividing, were found between normal acini—suggestive evidence for beginning invasion. Inside the growth the cells were arranged in form of alveoli, generally free of colloid, or in solid sheets enclosing occasionally a well formed acinus. This was the only thyroid tumour in this series the benign character of which was doubtful.

The two other females of the hooded strain (Rats 5 and 6) showed similar changes in pituitary and thyroid. They were 26½ and 29 months old when sacrificed. In the older the thyroid contained a whitish oblong nodule meauring 3 × 1 mm. at the surface. The pituitary was of irregular shape with a small haemorrhagic brownish area. This was a well defined basophil adenoma, the tumour cells giving the same staining reactions as described above. The thyroid nodule resembled the adenoma depicted in Fig. 11. The neoplastic changes found in the hypophysis and thyroid of the younger animal were of microscopic size. In the slightly abnormally shaped anterior lobe numerous thyroidectomy cells and also multiple nodules formed by atypical basophils were present, the pars intermedia being normal. The well stimulated thyroid contained a small adenoma of the solid type.

*Rats* 7 *and* 8.

Rat 7, a male of the Wistar strain, was sacrificed at the age of 25½ months, about 12 weeks after KI had been added to the drinking water. This was an apparently healthy animal weighing 292 g. At the post-mortem examination the pituitary was found to be symmetrically enlarged (67·4 mg.) and of whitish colour. On cutting the hypophysis appeared solid and free from haemorrhagic discoloration commonly seen in pituitaries of such size. Of the other endocrine glands only the suprarenals (39·6 mg.) differed from the normal picture by their darker colour. The sex organs (testis 2·8 g.) were of normal size and the thyroid was rather pale (25·2 mg.). The lungs were emphysematous and contained a small area of consolidation.

Multiple sections of the pituitary revealed that the bulk of the gland was tumourous. Normal anterior lobe tissue was recognisable in the periphery as a narrow rim surrounding approximately half of the circumference of the tumour. Radiating from this rim compressed strands of predominantly acidophilic and chromophobic elements were seen between areas of tumour, an indication that the neoplasm had arisen from different foci. Intermediate and posterior lobes appeared normal. The cells composing the tumour were large, showed great variation in size and shape, had sharply defined cell borders and vesicular nuclei with one or more large fuchsinophilic nucleoli. Multi-nucleated and tumour giant cells were rather numerous and so were mitoses. When stained by the PAS technique or with Gomori's aldehyde fuchsin after long search an occasional cell was found having PAS or Gomori positive granules. Only in this respect, did the adenoma differ from those described above. In Papanicolaou preparations the cytoplasm stained faintly with light green.

The histology of the thyroid was that of an inactive gland. The alveoli were lined by a low cuboidal epithelium and had ample colloid. One lobe contained an adenoma of follicular structure. Here the epithelium was also cuboid, the cells being more crowded and having nuclei much richer in chromatin than in the rest of the gland. Another feature of this well defined nodule were cystic dilated acini filled with colloid.

An unexpected finding was the presence of a tumour of microscopic size situated in the medulla of the left suprarenal. The cortex appeared normal as to cytology and distribution of lipoid but in the medulla a nodule was present, formed by cells resembling the normal chromaffin elements except for the scantiness of the cytoplasm and the increased chromatin content of the nuclei. Many mitoses were found in this nodule, considered to be a benign pheochromocytoma.

Rat 8, a 26 months old male, was killed because of the presence of an abscess in the subcutaneous tissue of the neck. Otherwise the animal was in an excellent state of health and weighed 460 g. when sacrificed. This rat had recieved KI supplement in the drinking water during the last 7 months of life. No obvious changes were seen in any of the internal organs at the post-mortem examination, but histological study of thyroid and pituitary revealed the presence of a centrally situated adenoma in the former and an aggregation of basophils in the latter. As in Rat 7, the thyroid showed a picture of a resting gland and the cells forming the adenoma were arranged in an alveolar pattern and were of low cuboid shape (Fig. 15). The only abnormal feature in the pituitary was a region in which basophils of the thyrotroph type predominated. These cells differed in three respects from the elements forming the pituitary tumours so far described. They varied relatively little in size snd shape, mitoses were absent and nearly all gave a strong reaction for glycoprotein (Fig. 14), in other words they resembled more normal than neoplastic thyrotrophs. This aggregation of basophils had no sharp boundaries nor did it compress the surrounding tissue, but large sinuses well filled with erythrocytes were seen in its centre so that the blood supply here was superior to that of the remaining pars anterior.

### DISCUSSION

Before entering into a discussion of the pathological observations recorded in this paper some fundamentals of the histology of the normal pituitary have to be examined. The first question to be answered is : which cells produce the thyrotrophic hormone and the second, how many types of basophils occur in the anterior lobe of the hypophysis. As to the first, most authors seem now to concur that this hormone is produced by basophilic cells. Thyrotrophin is a glycoprotein. The acidophils of the pituitary of the rat do not give a positive reaction for glycoprotein whereas the basophils do. Thus, cytochemical evidence indicates the production of the thryotrophic hormone by the latter (Purves and Griesbach, 1951*a* ; Pearse, 1952*a* and 1952*b*). As to the second, Romeis (1940) described two types of basophils both stainable with aniline blue but distinguishable by their affinity to creso-fuschin or resorcinol-fuschin. The type stained by these fuchsin dyes is the beta cell, the other is the delta cell of Romeis. As shown by Halmi (1950) another elastica stain, Gomori's fuchsin aldehyde reagent, also differentiates between two types of basophils, whereas most other techniques like Papanicolaou's or Gram's method stain all basophils. Pearse (1952*a*) does not seem to attach great significance to these different tinctorial qualities and adheres to a unitarian view. The older literature contains additional evidence in support of the contention that two types of basophils exist in the anterior lobe, evidence mainly derived from the study of the morphology and behaviour of the so-called thyroidectomy and castration cells (Zeckwer, 1937, 1938; Reese, Koneff and Wainman, 1943). More recently Purves and Griesbach (1951*a*, 1951*b*, and 1951*c*) by correlating morphological evidence based on modern cytochemical methods with the results obtained by bio-assay of the hormone content of the gland, and with the reaction of the pituitary cells to physiological amounts of oestrogen and of thyroxine, have, in the writer's opinion, offered overwhelming evidence for the existence of at least two kinds of basophils for which they suggested the names "gonadotrophs" and "thyrotrophs." These recent advances in our knowledge of morphology and function of the normal basophilic cells are of considerable help to the student of the chromophilic tumours of the pituitary. One is on less certain ground when dealing with the pathology of the pars intermedia. Two of the seven tumours presented in this communication show an intimate relation with the pars intermedia and similar observations have been recorded in the literature (Fischer, 1926; Rasmussen and Nelson, 1938). It seems, therefore, justifiable to review briefly those aspects of the histology of the pars or zona intermedia which are relevant to the problem in hand. Romeis (1940) confirming Rasmussen's (1930) observations, states that the basophils found in the intermediary zone of the human pituitary originate from the epithelium forming the posterior wall of the hypophyseal cavity. Only in this respect do they differ from the beta cells of the anterior lobe, with which they share the affinity for creso-fuchsin, resorcinol-fuchsin and other dyes staining these cells specifically. The question whether the difference in histogenesis is of functional significance was left open by Romeis (1940). According to Herring and Biedl (quoted from Romeis, 1940, p. 535) thyroidectomy leads to an increase in the size of the intermediary lobe in dog, cat and rabbit. Not infrequently one finds in rats treated with goitrogens for prolonged periods a pars intermedia which appears hyperplastic (Malcolm, Griesbach, Bielschowsky and Hall, 1949). Also in some species the pars intermedia proliferates when stimulated by oestrogens (Vazquez-Lopez, 1944). These observations suggest that some stimuli to which the cells of the anterior lobe react with an increase in their numbers affect in a similar manner elements of the intermediate lobe. It has however, to be remembered that in the rat the cells of the intermediary lobe differ in shape from the thyrotrophs of the anterior lobe, with which they share the affinity for the PAS stain and for Gomori's fuchsin aldehyde reagent.

In the preceding paragraphs the work has been reviewed which led to the conception of a special type of basophil concerned with the production of thyrotrophic hormone. In the experimental part when describing the pituitary adenoma of Rat 1, the similarity of the tumour cells with the thyrotrophs of the normal pituitary has been streesed and the opinion expressed that the tumour is formed by neoplastic thyrotrophs. Now the pathogenesis of the basophil tumours can be discussed. Chronic iodine deficiency lowers the thyroxine level to which the pituitary responds with an increase in thyrotrophic cells. In time, this compensatory hyperplasia progresses towards the formation of tumours, which in some instances reach considerable size. In this connection it might be mentioned that proliferating normal thyrotrophs, as observed by Guyer and Claus (1937), tend to aggregate in clusters, a mode of growth which might be favourable to nodular hyperplasia, the first stage in adenoma formation. On the other hand proliferating gonadotrophs tend to spread in a more diffuse manner, and so far tumours derived from them have not been observed in aged gonadectomised rats.

When considering abnormal growth in the pituitary of the rat one has to distinguish between true neoplasia and conditioned growth. The remarkable enlargements of the hypophysis seen after chronic administration of high amounts of oestrogen regress when treatment ceases. The basophilic adenomata, however, are considered to be true neoplasms, because the tumour found in the pituitary of Rat 7 showed all signs of active growth long after the stimulus which led to its formation had been removed by the administration of KI. The adenoma of this animal was functionally inactive as judged by the appearance of the thyroid which was that of a resting gland. Also cells giving a positive reaction for glycoprotein were virtually absent in this tumour. The most convincing evidence for functional activity is provided by Rat 4. The large pituitary tumour of this animal contained numerous PAS and Gomori positive elements and its thyroid showed the most pronounced signs of stimulation. In addition, in the pituitary of this animal, so little normal anterior lobe tissue was left that it seems most unlikely that this small rest could have secreted amounts of thyrotrophin large enough to induce a four-five fold enlargement of the thyroid. Finally the pituitary lesion of Rat 8 has to be considered. In this case the morphological evidence was more in favour of hyperplasia than of neoplasia. Here an agglomeration of thyrotrophs had persisted in one area despite the fact that KI had been supplied for 7 months. In the rest of the anterior lobe these cells were found in normal numbers, as was to be expected, and the thyroid had undergone involution. The rich glycoprotein content of the cells forming this aggregation was probably due to storage and not a sign of secretory activity. The anomalous behaviour of these cells suggests that they were no longer normal thyrotrophs.

The thyroid tumours observed are, in the writer's opinion, due to stimulation by elevated amounts of thyrotrophic hormone. The stimulus for the increased secretion by the pituitary was a deficiency of thyroxine due to impaired synthesis by an inadequate content of iodine in the diet. In six instances the thyroid tumours were found in activated and twice in resting glands. The latter occurred in animals which had received a supplement of KI for several months and are considered to be evidence of past stimulation. These neoplasms differed morphologically from those observed in stimulated thyroids in the same way as the thyroid adenomata found during administration of thiouracil differ from those seen after the treatment with the goitrogen has ceased. Their epithelium was rather low, and cystic follicles filled with colloid were a prominent feature. To summarise the pathogenesis and structure of the thyroid adenomata observed is similar to that of the tumours induced by goitrogens which act by blocking the synthesis of thyroxine.

The conception that thyroxine deficiency is the ultimate cause of the syndrome described is strengthened by the following observations. Fischer (1926), writing from Bern, Switzerland recorded two cases of pituitary tumours found in aged female rats. The thyroids of both animals contained multiple tubular adenomata, and in one an adenocarcinoma was also present in this gland. The rats belonged to Wegelin's colony in which goitre was endemic Fischer's paper was published long before the gonadotrophic and thyrotrophic hormones had been discovered. She stressed, however the similarity of the pituitary tumour cells with the elements which appear in the rat's hypophyis after castration or thyroidectomy; and considered the neoplasms to be carcinomata formed by " castration " cells although the ovaries of one of her animals were normal. Fischer believed that the neoplasms originated from the pars intermedia or tuberalis. " Spontaneous " tumour formation occurring simultaneously in thyroid and pituitary of rodents seems to have been observed only by Fischer and by the writer, but one sees this syndrome under experimental conditions most frequently in animals treated with goitrogens for prolonged periods. So Purves and Griesbach (1951*a*) mention a basophil adenoma of a rat treated with methythiouracil for two years. Basophil adenomata occur also in thyroidectomised rats treated with acetylaminofluorene (unpublished results). Into the same class belong also the pituitary tumours discovered by Gorbman (1950). They appear in mice exposed to high doses of radioactive iodine, but can be prevented by the administration of thyroxine (Goldberg and Chaikoff, 1951 ; Gorbman, 1952). Furth, Gadsden and Upton (1952) reported that transplants of such tumours contain large amounts of thyrotrophic hormone. The nature of the basophil adenomata found in gonadectomised mice of certain strains (Dickie and Woolley 1949) has not yet been established. In such mice adrenal cortical neoplasms develop in consequence of the removal of ovaries or testes early in life. It is not claimed that all basophil adenomata of the hypophysis are derived from thyrotrophs, but it might be pointed out that high doses of cortisone suppress thyroid function, and that the changes seen in the pituitaries of patients suffering from Cushing's disease could be due to this mechanism. Finally, of the vast literature on basophil adenomata in men, one example may be quoted. Rasmussen and Nelson (1938) described two cases in which basophil tumours arose in the intermediary lobe, and in both patients adenomata were found also in the atrophic thyroid. Rasmussen's publication comes from Minneapolis, a town situated in a region of the U.S.A. where goitre is endemic, and where according to Rice (1938), in the higher age-groups most thyroids are adenomatous. It is therefore possible that the coincidence of neoplastic changes in the two endocrine glands was due to chance.

### SUMMARY.

Functional and non-functional basophil adenomata of the pituitary in conjunction with adenomata of the thyroid have been found in aged rats of two strains. These basophil adenomata are considered to be true tumours formed by neoplastic thyrotrophs.

Chronic thyroxine deficiency due to an inadequate iodine content of the diet is believed to be the cause of the syndrome. Chronic thyroxine deficiency leads to a compensatory increase in thyrotrophs and finally to neoplastic changes in the hypophysis, and long continued stimulation by elevated amounts of thyrotrophic hormone is the cause of hyperplasia and adenoma formation in the thyroid.

### REFERENCES.

Dickie, M. M., and Woolley, G. W.—(1949) *Cancer Res.*, **9**, 372.
Fischer, O.—(1926) *Virchows Arch.*, **259**, 9.
Furth, J., Gadsden, E. L., and Upton, A. C.—(1952) *Cancer Res.*, **12**, 739.
Goldberg, R. C., and Chaikoff, I. L.—(1951) *Endocrinology*, **48**, 1.
Gorbman, A.—(1950) *J. clin. Endocrin.*, **10**, 1177.—(1952) *Proc. Soc. exp. Biol., N.Y.*, **80**, 538.

GREEN, J. D.—(1951) *Amer. J. Anat.*, **88**, 225.
GUYER, M. F., AND CLAUS, P.E.—(1937) *Anat. Rec.*, **67**, 145.
HALMI, N. S.—(1950) *Endocrinology*, **47**, 289.
LILLIE, R. D.—(1952) *Amer. J. clin. Path.*, **21**, 485.
MALCOLM, J., GRIESBACH, W. E., BIELSCHOWSKY, F., AND HALL., W. H.—(1949) *Brit. J. exp. Path.*, **30**, 17.
PEARSE, A. G. E.—(1952*a*) *Ciba Foundation Colloquia on Endocrinology*, **4**, 1, 51.—(1952*b*) *J. Path. Bact.*, **64**, 811.
PURVES, H. D., AND GRIESBACH, W. E.—(1951*a*) *Endocrinology*, **49**, 244.—(1951*b*) *Ibid.*, **49**, 427.—(1951*c*) *Ibid.*, **49**, 652.
RASMUSSEN, A. T.—(1930) *Amer. J. Anat.*, **46**, 461.
*Idem* AND NELSON, A. A.—(1938) *Amer. J. Path.*, **14**, 297.
REESE, J. D., KONEFF, A. A., AND WAINMAN, P.—(1943) ‘Essays in Biology,’ p. 473. University of California Press.
RICE, C. O.—(1938) *Arch. Surg.*, **36**, 96.
ROMEIS, B.—(1940) *Handbuch der Mikroskopischen Anatomie des Menschen*, Vol. **6**, part 3. Berlin (Springer).
VAZQUEZ-LOPEZ, E.—(1944) *J. Path. Bact.*, **56**, 1.
ZECKWER, I. T.—(1937) *Amer. J. Path.*, **13**, 985.—(1938) *Ibid.*, **14**, 773.